# Supplementary material for: College students’ sense of belonging and alcohol use amidst COVID-19: Evidence from a 21-day daily diary study
Source: PLoS One. 2024 Dec 3;19(12):e0310496. doi: 10.1371/journal.pone.0310496 (PMC11614200; doi:10.1371/journal.pone.0310496)
Supplement: S1 File — (DOCX) [file pone.0310496.s002.docx]

**Supplemental Materials for**

**“****College Students’ Sense of Belonging and Alcohol Use amidst COVID-19: Evidence from a 21-day Daily Diary Study”**

*This supplement provides supplemental analyses beyond those included in the main manuscript.*

**Table S1. Mixed Models Examining Associations between Belonging Uncertainty and Alcohol Use Behaviors (By Race/Ethnic Categories)**

|  | Non-Hispanic Black | | Non-Hispanic Asian | | Hispanic | | |
| --- | --- | --- | --- | --- | --- | --- | --- |
|  | Any Alcohol Use | Number of drinks | Any Alcohol Use | Number of drinks | Any Alcohol Use | Number of drinks |  |
| Fixed effects |  |  |  |  |  |  |  |
| Level 1 |  |  |  |  |  |  |  |
| Daily belonging  uncertainty | -0.035^***^ | -0.166^***^ | -0.035^***^ | -0.166^***^ | -0.035^***^ | -0.166^***^ |  |
|  | (0.004) | (0.024) | (0.004) | (0.023) | (0.004) | (0.024) |  |
| Level 2 |  |  |  |  |  |  |  |
| Average belonging  uncertainty | -0.020^***^ | -0.136^***^ | -0.021^***^ | -0.135^***^ | -0.020^***^ | -0.138^***^ |  |
|  | (0.006) | (0.035) | (0.006) | (0.032) | (0.006) | (0.034) |  |
| Gender | -0.012 | -0.356^***^ | -0.006 | -0.291^***^ | -0.010 | -0.325^***^ |  |
|  | (0.011) | (0.064) | (0.010) | (0.056) | (0.010) | (0.061) |  |
| First generation | 0.009 | -0.021 | 0.009 | -0.045 | 0.011 | -0.026 |  |
|  | (0.014) | (0.080) | (0.012) | (0.072) | (0.013) | (0.076) |  |
| LGBTQ+ | -0.038^***^ | -0.243^***^ | -0.036^***^ | -0.224^***^ | -0.034^***^ | -0.239^***^ |  |
|  | (0.013) | (0.078) | (0.012) | (0.071) | (0.013) | (0.074) |  |
| Non-Hispanic Black | -0.097^***^ | -0.517^***^ |  |  |  |  |  |
|  | (0.029) | (0.168) |  |  |  |  |  |
| Non-Hispanic Asian |  |  | -0.101^***^ | -0.544^***^ |  |  |  |
|  |  |  | (0.012) | (0.070) |  |  |  |
| Hispanic |  |  |  |  | -0.033^**^ | -0.207^**^ |  |
|  |  |  |  |  | (0.016) | (0.093) |  |
| Cross-level Interactions |  |  |  |  |  |  |  |
| Daily Belonging  Uncertainty x Non-  Hispanic Black | 0.013 | 0.129 |  |  |  |  |  |
|  | (0.022) | (0.124) |  |  |  |  |  |
| Daily Belonging  Uncertainty x Non-  Hispanic Asian |  |  | 0.024^**^ | 0.107^*^ |  |  |  |
|  |  |  | (0.010) | (0.057) |  |  |  |
| Daily Belonging  Uncertainty x Hispanic |  |  |  |  | 0.016 | 0.079 |  |
|  |  |  |  |  | (0.013) | (0.076) |  |
| Random effects |  |  |  |  |  |  |  |
| Slope variance | 0.003^***^ | 0.078^***^ | 0.002^***^ | 0.071^***^ | 0.002^***^ | 0.091^***^ |  |
|  | (0.001) | (0.022) | (0.001) | (0.019) | (0.001) | (0.022) |  |
| Intercept variance | 0.025^***^ | 0.890^**^ | 0.024^***^ | 0.825^***^ | 0.025^***^ | 0.870^***^ |  |
|  | (0.001) | (0.043) | (0.001) | (0.037) | (0.001) | (0.041) |  |
| Residual variance | 0.113^***^ | 3.541^***^ | 0.105^***^ | 3.253^***^ | 0.114^***^ | 3.562^***^ |  |
|  | (0.001) | (0.034) | (0.001) | (0.029) | (0.001) | (0.033) |  |
| *N* | 23354 | 23337 | 27014 | 26997 | 25088 | 25070 |  |

*Note*. Standard errors in parentheses. * p < .10, ** p < .05, *** p < .01

**Table S2. Mixed Models Examining Associations between Belonging Uncertainty and Alcohol Use Behaviors (Day of week Controlled)**

| Dependent Variables | Any Alcohol Use | | | | | Total Number of Drinks | | | | |
| --- | --- | --- | --- | --- | --- | --- | --- | --- | --- | --- |
| Fixed effects | (1) | (2) | (3) | (4) | (5) | (1) | (2) | (3) | (4) | (5) |
| Level 1 |  |  |  |  |  |  |  |  |  |  |
| Daily Person-centered Belonging Uncertainty | -0.021^***^ | -0.015^**^ | -0.007 | -0.020^***^ | -0.023^***^ | -0.098^***^ | -0.090^***^ | -0.033 | -0.092^***^ | -0.107^***^ |
|  | (0.003) | (0.006) | (0.006) | (0.003) | (0.003) | (0.018) | (0.034) | (0.033) | (0.019) | (0.020) |
| Level 2 |  |  |  |  |  |  |  |  |  |  |
| Average person-mean Belonging Uncertainty | -0.019^***^ | -0.019^***^ | -0.019^***^ | -0.019^***^ | -0.019^***^ | -0.121^***^ | -0.121^***^ | -0.121^***^ | -0.121^***^ | -0.121^***^ |
|  | (0.005) | (0.005) | (0.005) | (0.005) | (0.005) | (0.028) | (0.028) | (0.028) | (0.028) | (0.028) |
| Woman | -0.001 | -0.001 | -0.001 | -0.001 | -0.001 | -0.232^***^ | -0.232^***^ | -0.232^***^ | -0.232^***^ | -0.232^***^ |
|  | (0.009) | (0.009) | (0.009) | (0.009) | (0.009) | (0.050) | (0.050) | (0.050) | (0.050) | (0.050) |
| Minoritized  Student | -0.075^***^ | -0.075^***^ | -0.075^***^ | -0.075^***^ | -0.075^***^ | -0.411^***^ | -0.411^***^ | -0.410^***^ | -0.411^***^ | -0.410^***^ |
|  | (0.009) | (0.009) | (0.009) | (0.009) | (0.009) | (0.051) | (0.051) | (0.051) | (0.051) | (0.051) |
| First generation  Student | 0.010 | 0.010 | 0.010 | 0.010 | 0.010 | -0.052 | -0.052 | -0.052 | -0.052 | -0.052 |
|  | (0.011) | (0.011) | (0.011) | (0.011) | (0.011) | (0.062) | (0.062) | (0.062) | (0.062) | (0.062) |
| LGBTQ | -0.029^***^ | -0.029^***^ | -0.029^***^ | -0.029^***^ | -0.029^***^ | -0.203^***^ | -0.203^***^ | -0.203^***^ | -0.203^***^ | -0.203^***^ |
|  | (0.011) | (0.011) | (0.011) | (0.011) | (0.011) | (0.063) | (0.063) | (0.063) | (0.063) | (0.063) |
| Cross-level interactions |  |  |  |  |  |  |  |  |  |  |
| Daily Belonging Uncertainty x Woman |  | -0.009 |  |  |  |  | -0.011 |  |  |  |
|  |  | (0.007) |  |  |  |  | (0.039) |  |  |  |
| Daily Belonging Uncertainty x Minoritized |  |  | 0.020^***^ |  |  |  |  | 0.092^**^ |  |  |
|  |  |  | (0.007) |  |  |  |  | (0.039) |  |  |
| Daily Belonging Uncertainty x First generation |  |  |  | -0.009 |  |  |  |  | -0.036 |  |
|  |  |  |  | (0.008) |  |  |  |  | (0.046) |  |
| Daily Belonging Uncertainty x LGBTQ |  |  |  |  | 0.007 |  |  |  |  | 0.046 |
|  |  |  |  |  | (0.008) |  |  |  |  | (0.044) |
| Random effects |  |  |  |  |  |  |  |  |  |  |
| Slope variance | 0.001^***^ | 0.001^***^ | 0.001^***^ | 0.001^***^ | 0.001^***^ | 0.058^***^ | 0.058^***^ | 0.058^***^ | 0.058^***^ | 0.057^***^ |
|  | 0.000 | 0.000 | 0.000 | 0.000 | 0.000 | (0.014) | (0.014) | (0.014) | (0.014) | (0.014) |
| Intercept variance | 0.024^***^ | 0.024^***^ | 0.024^***^ | 0.024^***^ | 0.024^***^ | 0.798^***^ | 0.798^***^ | 0.798^***^ | 0.798^***^ | 0.798^***^ |
|  | (0.001) | (0.001) | (0.001) | (0.001) | (0.001) | (0.032) | (0.032) | (0.032) | (0.032) | (0.032) |
| Residual variance | 0.089^***^ | 0.089^***^ | 0.089^***^ | 0.089^***^ | 0.089^***^ | 2.707^***^ | 2.707^***^ | 2.706^***^ | 2.707^***^ | 2.707^***^ |
|  | (0.001) | (0.001) | (0.001) | (0.001) | (0.001) | (0.023) | (0.023) | (0.023) | (0.023) | (0.023) |
| *N* | 31422 | 31422 | 31422 | 31422 | 31422 | 31402 | 31402 | 31402 | 31402 | 31402 |

*Note*. Standard errors in parentheses. * p < .10, ** p < .05, *** p < .01. Day of the week (dummy variables for each day with Sunday serving as reference group) included as a level-1 fixed effect in above models. All coefficients are statistically significant at and negative in magnitude showing relatively lower alcohol use on days vis-à-vis Sunday; not shown in table above for ease of interpretation and comparison with main Table 3.
