# Supplementary material for: College students’ sense of belonging and alcohol use amidst COVID-19: Evidence from a 21-day daily diary study
Source: PLoS One. 2024 Dec 3;19(12):e0310496. doi: 10.1371/journal.pone.0310496 (PMC11614200; doi:10.1371/journal.pone.0310496)
Supplement: Attachment — Submitted filename: PONE-D-23-38054 .docx [file pone.0310496.s003.docx]

PONE-D-23-38054

Thank you very much for giving me the opportunity to review this article. I found the topic quite interesting, and a very important issue to consider when working with college students. This kind of investigations are necessary to improve the passage of undergraduates through the college years. Although it is very well documented, I have some concerns with the way in which the study has been approached. I have the impression that the approach and the analyses do not coincide with the discussion of the results, as if there were two different objectives.

**Introduction:**

p. 3 “Given that major differences exist in the impact of COVID-19 on health outcomes by gender, underrepresented racial and ethnic minorities status (URM), first-generation status, and LGBTQ status” How is it? It would be beneficial for the introduction to specify the patterns and the results of the different studies, to better understand which the differences are.

It would be interesting to describe how the situation was in 2021 when the study was implemented. There are countries, such as Spain, where the measures and policies were very strict by then (curfews, limitations on being at the street, limitations on group reunions, etc.). These specific situations may have had an impact on the alcohol use tendencies in college students. If students had to share more time in campus because of some specific policy, maybe it led to a higher sense of belonging, and vice versa. For example, during 2021, in some Spanish universities, students only had to be one of every three weeks present in classes, with the 2 other weeks following remote lessons. This situation irremediably must affect the sense of belonging, particularly in those that 2021 was their first college year. The authors include some reflections in the discussion about this issue, but I feel that it would be positive to address it in the introduction also.

**Method and analyses**

It is no clear for me if the research questions included where or with whom the participants drank, because it is not the same to drink alone that to meet some friends or colleagues to have some drinks. I believe that this is also an interesting point to consider in this investigation, because I assume that the way and the people you drink with affect in any way to the sense of belonging.

**Discussion**

Also, although the discussion is very well conducted, and the analyses show interesting results, I got confused during the reading of the manuscript because I do not know if the results were as expected or were completely different. I know that the research questions were mostly descriptive, and that the study design responds to them, but for me was unexpected the result “a higher uncertainty in belonging is related to a less likely of consumption of alcohol”. It is true that the authors explain this result in the discussion, but in the introduction, I could not find information related to this possible result, or to the contrary. In fact, it seems that the main result (that URM population had the opposite behavior than the other groups) has nothing to do with the objective of the study (because it is no mentioned that this result can be achieved).

It is clear that alcohol consumption can be problematic in university students, and that university institutions must address this issue; however, I believe that the future lines of investigation are also scarce. There is potential in the study results that I believe is not being addressed. On the one hand, to implement measures of welcome, of belonging that do not involve alcohol consumption (since, according to the study, the greater the feeling of belonging, the greater the alcohol consumption). On the other hand, what can be done with minority populations (who happen to be the ones who consume more alcohol when they feel less belonging)?

For all these reasons, I consider that the article should be rewritten, emphasizing in the introduction the different points that I have tried to highlight in the review.
