# Supplementary material for: College students’ sense of belonging and alcohol use amidst COVID-19: Evidence from a 21-day daily diary study
Source: PLoS One. 2024 Dec 3;19(12):e0310496. doi: 10.1371/journal.pone.0310496 (PMC11614200; doi:10.1371/journal.pone.0310496)
Supplement: Attachment — Submitted filename: PONE-D-23-38054_reviewer comments.docx [file pone.0310496.s004.docx]

**College Students’ Sense of Belonging and Alcohol Use amidst COVID: Evidence from a 21-day Daily Diary Study**

Overall, the manuscript provides valuable insights into the linkages between a key psychosocial factor—students’ sense of belonging in college—and their alcohol use patterns amidst COVID. The manuscript's findings hold significant value for mental health care and public health services. It is well-written and organized, with a clear introduction that sets the context for the study. However, implementing the recommendations would further improve the clarity, robustness, and applicability of the findings.

**General recommendation:**

1. Although the language is generally clear, improving sentence structure, selecting better words, and ensuring coherence could enhance readability. Additionally, it is recommended to proofread for grammatical and typographical errors.
2. The reference style does not meet the journal's requirements, and it should adhere to the Vancouver style.

**More specific recommendation:**

**introduction:**

The introduction is well-written, concise, and clear, effectively highlighting the gap in the literature and explaining the aim of the present study. The heading for the current study at the end of the introduction is provided very effectively.

**Method:**

In this section, it is advisable to introduce the study's methodology before delving into the sampling and population. This could include specifying whether the study is qualitative or quantitative, descriptive or applied, and whether it involves multivariate correlation analysis, among other relevant details.

Participant’s part is clear, however:

- Table 1, labeled as "Descriptive Statistics" in this section, should be renamed to avoid misleading the reader. This is because Table 2, titled as "Descriptive Statistics of Participants," also appears in the results section, and it's important to distinguish between the two.
- The paragraph that begins with "In all, 2,068 participants consented and participated in the Student Engagement…" should be titled as "Procedure" as a subheading in the Method section.

**Results:**

The results section offers a comprehensive presentation of relevant statistical analyses. The tables effectively convey important data necessary for interpreting the results, and the author skillfully avoids redundancy and unnecessary elaboration within the tables.

- However, the titles of the tables need to be more specific and clear for readers.

**Discussion:**

The discussion part effectively explores the study findings and comparing them with previous research. It appropriately addresses the limitations of the study and suggests avenues for future research.

- It is recommended that the last paragraph of the discussion part be titled "Conclusion" to provide clarity for readers.
- In the last sentence of paragraph 2, which starts with "These findings are in line with prior patterns observed in the literature from cross-sectional…", the reference is missing.
