# Supplementary material for: College students’ sense of belonging and alcohol use amidst COVID-19: Evidence from a 21-day daily diary study
Source: PLoS One. 2024 Dec 3;19(12):e0310496. doi: 10.1371/journal.pone.0310496 (PMC11614200; doi:10.1371/journal.pone.0310496)
Supplement: Attachment — Submitted filename: ResponseLetter_R1_Submitted.docx [file pone.0310496.s005.docx]

June 25, 2024

Dear Editors:

Thank you for your invitation to resubmit our manuscript, “College Students’ Sense of Belonging and Alcohol Use amidst COVID: Evidence from a 21-day Daily Diary Study”. We are grateful to you and the reviewers for your thoughtful consideration of our work and your helpful suggestions. We were delighted to hear comments such as, *“...Overall, the manuscript provides valuable insights into the linkages between a key psychosocial factor—students’ sense of belonging in college—and their alcohol use patterns amidst COVID. The manuscript's findings hold significant value for mental health care and public health services. It is well-written and organized, with a clear introduction that sets the context for the study”* Most of the reviewers’ comments called for clarification and further elaboration of methods and results in the main manuscript. We have used these suggestions to guide our revision. We believe that the revision fully addresses all concerns raised and is a stronger manuscript.

In the sections that follow, we respond to each reviewer’s concerns (shown in italics) in turn. In our responses, we indicate the specific changes we made and, as appropriate, point to relevant pages and tables; we have also used track changes in the revised version of the manuscript.

Again, we are grateful for the opportunity to resubmit a revised version of this manuscript to *PLOS ONE*. We look forward to your response.

Sincerely,

Authors

# ***Responses to Comments by Reviewer 1***

*Overall remarks: This article presents the relationship between daily sense of belonging to a campus for college students and their alcohol consumption during the COVID-19 pandemic. The authors demonstrated that more uncertainty regarding sense of belonging to the college campus is related to lower alcohol consumption. Underrepresented racial minorities and student identifying to the LGBTQ community reported more uncertainty with their belonging, which was related to higher alcohol use. This article is overall well written and uses appropriate statistical methods to respond to their research questions. However, it is not publishable in its current state. Throughout this article, slight modifications in the phrasing and corrections could improve dramatically its clarity. Also, content belonging in the results section is presented in the methods and should be moved. Furthermore, methodological issues are not addressed properly and could benefit from transparency and a deeper discussion in the methods or in the limitations section. Finaly this article seem[s] relevant and could be of interest for the readers of PLOS One.*

1. *Abstract. The abstract is short and present a good overview of the content of this manuscript. However, slight modifications could greatly improve its quality.*
   a. *What to you refer to when talking about sense of belonging? To what institution or group’s belonging are you measuring in this article?*

Thank you for highlighting this. We have revised the abstract now to clarify that we are examining students’ sense of belonging to their college institution in this article.

b. *The phrasing of the result section could be improved as the first sentence can be hard to understand at first glance.*

We have now slightly edited the first sentence in the results section to clarify that “more uncertain about belonging” refers to “lower belonging” in parentheses. Even if a bit complex, *belonging uncertainty* is the key measure we use in this study. So, we wanted to be precise in the use of this terminology throughout. However, to ease readability and interpretation of key results in the abstract, we added this clarification. HoHo

2. *Introduction. The introduction offers a good overview of the current literature and introduces well the concepts used in this manuscript.*
   a. *However, it could benefit from a little introduction to the definition of the sense of belonging and of measures often used to measure it. This could offer a good ground for comparison with the measure used in this project*

We have now added the key definitions/items of belonging used in other studies that we cite in the “Introduction” in parentheses and footnotes, wherever applicable (see pages 4 and 5).

b. *This sentence in the second paragraph is not clear: “Specifically, while some studies found that college students reported heavier use during relative to prior to the pandemic […]” are you referring to before the pandemic? At the beginning of the pandemic?*

We have now edited this sentence for more clarity. See here for quick reference: “Research on alcohol use patterns during the initial onset of the pandemic (i.e., early to mid 2020) revealed mixed patterns and trends. Specifically, while some studies found that college students reported heavier use during the pandemic (relative to prior to the pandemic) (3,4), others found that young adults might be drinking *less* overall, but instead drinking more frequently, and primarily with their family and/or alone (5,6).”

c. *The justification for the inclusion of sexual diversity, gender diversity and racial diversity is short, could you add a bit more context for the audience to better understand why these dimensions are important? Most of these populations are at risk for mental health disorder, but is it the case for alcohol use and abuse?*

Thank you for the suggestion! We have now added two sentences with relevant citations to the introduction: “Given that major differences exist in the impact of COVID-19 on health outcomes by gender, minoritized student status, first-generation status, and LGBTQ status (8–11), examining heterogeneity in alcohol use patterns across various college student subgroups amidst COVID-19 remains important. For example, increases in alcohol frequency, heavy drinking, and alcohol-related harms were observed only in women (12), and the link between psychological distress and alcohol use and use severity was also observed only in women (13,14). Lastly, during the pandemic individuals who identify as LGBTQ+ have been at disproportionate risk for mental health concerns and substance use (15,16).”

d. *This affirmation at page 4 needs a citation: “On the prevention side, a key emerging protective factor against adverse mental health outcomes—such as anxiety and depression, which is often a precursor to alcohol use […]”*

We have added a citation for this and slightly modified the sentence to be more accurate. We now state: “On the prevention side, a key emerging protective factor against adverse mental health outcomes—such as anxiety and depression, which often co-occur with alcohol use (e.g., Castillo -Carniglia et al., 2019 )…”

e. *This sentence in page 4 is a bit of an overstatement: “Students’ sense of belonging with their campus, often considered a fundamental human motivation (Baumeister & Leary, 1995) […]” the provided reference addresses sense of belonging at large, not something as specific as belonging to a specific campus. You can extend the importance of belonging to a college campus but please rephrase that. The rest of the sentence is really good and could support this extension.*

We have now removed “Students’” and “with their campus” in this sentence to accurately characterize belonging more generally mentioned in the cited study.

f. *This sentence in page 4 has no citation to support it: “For example, college students who reported feeling a strong sense of belonging with their institution prior to the onset of the pandemic reported lower levels of depressive symptoms and anxiety during the pandemic, even after controlling for their baseline (pre-COVID) depressive and anxiety symptoms.”*

We have added the citation again at the end of the paragraph to improve clarity. The full citation is included here for quick reference: Gopalan M, Linden-Carmichael A, Lanza S. College Students’ Sense of Belonging and Mental Health Amidst the COVID-19 Pandemic. *J Adolesc Health*. 2022 Feb;70(2):228–33.

g. *This sentence in page 5 is very interesting: “First, there is consistent evidence of sociodemographic differences by race/ethnicity, gender, and first-generation college student status in students’ sense of belonging.” but could you give a little bit more information about those results as this could offer already an idea of the direction of your results despite its longer distance between measures.*

Thank you! We have now added this sentence to immediately follow the sentence referred to above. “For example, women report higher sense of belonging with their campus than men, first-generation college students and minoritized students often report feeling lower belonging than their white and/or continuing-generation peers, especially in predominantly white institutions (19,21,23,33).”

h. *This section could benefit greatly from a slight introduction to the concept of belonging, a definition and a quick presentation of the measures often used to quantify this concept. The addition of a footnote in the methods section offers an important clarification of the method used but personally raised more questions regarding this measure, it’s quality and the actual concept measured here.*

We agree! By incorporating the earlier suggestions of including the definitions of belonging used in the prior literature and the addition of a footnote that clarifies the face validity of the measure we use in our study (see pages 4 & 5 and especially footnote i), we believe we have addressed this comment as well. We will be happy to add additional notes if the editor/reviewer feels strongly as well.

3. *Methods. The outline of this section is good, and it is overall well written. Nonetheless the presentation of measures should be dramatically improved for transparency and address eventual methods limitations in the study design and/or statistical analyses.*
   a. *I appreciate the transparency regarding ethical requirements in the PLOS ONE form, but could you please add a small statement in the methods section stating the university that you refer to in this sentence page 7: “The university’s Institutional Review Board approved the current study.*

Unfortunately, we do not have permission to name the “university” in the paper itself. As we agree that context of the university is important, we have noted that the four-year, public university in which our current study was conducted is located in the Northeast region of the US. We have added one more sentence regarding the ethical consideration for observing consent in this study as well in this version that we hope eases the reviewers’ concern.

b. *Descriptive statistics, including table 1 and the paragraph following it before the Measures section should belong in the results section (i.e. “While the analytical sample had a slightly larger proportion of female students than the broader university population (68.5% vs. 47.3% in population), the racial-ethnic composition of the sample (7.8% Hispanic, 2.5% non-Hispanic Black, 70.5% non-Hispanic White, 14.4% non-Hispanic Asian, and 4.7% other/mixed races) was largely similar to that of the population of students at this campus (7.7% Hispanic, 4.3% non-Hispanic Black, 65.2% non-Hispanic White, 6.7% non-Hispanic Asian, and 16.1% other/mixed races). For further details on the Project SELF protocol, please see Lanza and colleagues’ protocol (under review).”*

We have now renamed the table titles to make clear distinctions. Because Table 1 provides the descriptive statistics primarily of the analytic sample (i.e., the participants) we included it in this section immediately following our description of the participants. We included Table 2 which provides the descriptive statistics of the key variables in the analytic sample in the “Results” section. It is also renamed “Means and SDs of Key Variables (Disaggregated by Demographic Subgroups)” to clearly distinguish from Table 1. We are happy to re-consider the placement of the tables if the reviewer feels strongly. But we believe that this renaming of tables and placement helps the narrative flow of the paper significantly.

c. *The measure of belonging uncertainty is interesting as it seems to capture a large portion of the internal perspective of the student on its situation. It is nonetheless very limited as belonging to a college can have many layers such as belonging to specific groups within this institution, agreeing with this college’s administrative choices, etc. I do not reject this measure but as evoked in the introduction section, I would really like to see more regarding the definition of sense of belonging and addressing this small variable’s eventual limitations in this regard.*

As described above, we have now included definitions of belonging used in most other studies we cite in the introduction in this revised version. We also include in a footnote how the measure we use in our study is closely aligned with our question, study design, and is closely adapted from other extensively used measures/items of belonging.

d. *The measure of alcohol used alone or in a social setting is not presented here but included in the analytic plan which is rather confusing.*

We have now removed all references of “drinking alone” in this manuscript. The base rate of students who drank alone was extremely low in this sample (Person level *N* = 4). Hence, it did not make sense to use this outcome measure in our analysis even though we considered this earlier given the theoretical interest in assessing linkages between sense of belonging with others/campus to isolation and likely solitary drinking behavior vis a vis social drinking. We apologize for this confusion.

e. *The sociodemographic factors used are on one hand relevant and interesting but on the other very limited and incoherent, especially regarding the LGBTQ status. You add a measure regarding LGBTQ status but only offer two choices regarding gender, which is rather antithetic. The LGBTQ status refer to sexual and gender-diversity which are very different dimensions that can have major differences in the observed dynamics. When looking at the provided footnote, this classification as LGBTQ or non-LGBTQ seems to be rather a statistical choice rather than a methodological flaw. This is the same thing for racial groups. The measures in the methods section should be presented raw, as the variables were collected in the design, not as you chose to include it in your statistical analysis. The statistical choices should be provided after the presentation of the measures, so the overall design is totally transparent and your statistical choices too. The lack of diversity in your final sample can be interpreted as a limitation and your choice to group them to curb this limitation can be relevant but transparency is extremely important here. Therefore, I highly recommend reviewing thoroughly this section for us to understand if the limitation regarding sex and gender diversity is a methodological flaw in the study design, or rather a voluntary choice to balance statistical power and your research objectives.*

We completely agree with this reviewer and appreciate this comment. First, we now present the measures (and means/SDs) in raw form wherever possible without sacrificing ease of interpretation. See also Table A1 in appendix that breaks down the model by race/ethnicity subgroups. That said, we acknowledge the limitation of grouping all LGBTQ+ students in one category. We have now included this statistical power-based limitation in this version. See here for quick reference:

“Third, despite our large sample size (both person- and day-level) overall in the analytic sample, disaggregation by finer-grained race/ethnic, or LGBTQ subgroups is constrained by statistical power. Finally, although we use an extensively studied measure of belonging uncertainty in college student samples (see 19,21,22,33), we cannot rule out the limitations related to the measurement challenges inherent in belonging assessments used in the literature (43).”

f. *I appreciate the inclusion of an appendix table to present dynamics in racial subgroups. The analytic plan is clear and seem appropriate to respond to your questions.*

Thank you!

4. *Results. The results are overall well presented and seem complete. However, as your sense of belonging variable is reversed, it is a bit more complicated to understand the direction of results.*

We understand this concern and have added notes (i.e., that higher uncertainty about belonging is equivalent conceptually to lower belonging) where appropriate to ease readability and interpretation. In a daily diary study context where state-measures of belonging are being captured, we believe that the “uncertainty” framing of this measure is both theoretically and empirically powerful as it captures a more “dynamic” aspect of belonging that is relevant to daily assessments and especially in relation to alcohol use for this population. This measure used in the current study has been adapted from extensively used, conceptually valid, measures of belonging as described throughout in this revised version.

5. *Discussion and conclusion:*
   a. *Did you control for the day of the week when measures were taken? As social events typically occur during weekends, this would be extremely important to measure as students going to an event organized by the college’s student associations for example could feel a higher belonging and consume more alcohol, which could mostly drive your observed effect.*

We thank you for this suggestion. We have now added day-of-the-week adjusted mixed models in the appendix. As can be seen, this covariate(s) addition does not affect the magnitude or sign of associations between belonging and alcohol use across all model specifications (see Appendix Table A3).

b. *This sentence needs a citation: “Finally, women, URM, first-generation, and LGBTQ students show slightly higher belonging uncertainty even on a day-to-day basis, largely replicating prior work.*

We have added citations for this sentence, included below with minor edits from earlier. See here for quick reference:

“Finally, minoritized, first-generation, and LGBTQ students show slightly higher belonging uncertainty even on a day-to-day basis, largely replicating prior work (20,24)”

20 - Murphy MC, Gopalan M, Carter ER, Emerson KTU, Bottoms BL, Walton GM. A customized belonging intervention improves retention of socially disadvantaged students at a broad-access university. Sci Adv. 2020 Jul 1;6(29):eaba4677

24 - Gopalan M, Brady ST. College Students’ Sense of Belonging: A National Perspective. Educ Res [Internet]. 2020 Mar 1 [cited 2020 Mar 3];49(2):134–7. Available from: https://doi.org/10.3102/0013189X19897622

c. *The limitation section should underline the limitations regarding LGBTQ and URM groups being the lack of fine measures of LGBTQ status and the grouping of URM groups due to small size as these can have an important influence on results and should be addressed in further studies.*

See our note above regarding how we have incorporated this now in the “Limitations” section of this revised version.

# ***Responses to Comments by Reviewer 2***

*Thank you very much for giving me the opportunity to review this article. I found the topic quite interesting, and a very important issue to consider when working with college students. This kind of investigations are necessary to improve the passage of undergraduates through the college years. Although it is very well documented, I have some concerns with the way in which the study has been approached. I have the impression that the approach and the analyses do not coincide with the discussion of the results, as if there were two different objectives.*

We thank Reviewer #2 for thoughtfully engaging with our work. Below we discuss how we address the remaining recommendations made by Reviewer #2

1. *Introduction: p. 3 “Given that major differences exist in the impact of COVID-19 on health outcomes by gender, underrepresented racial and ethnic minorities status (URM), first-generation status, and LGBTQ status” How is it? It would be beneficial for the introduction to specify the patterns and the results of the different studies, to better understand which the differences are.*

We agree. We have now added these sentences to the introduction. Included here for quick reference: “Given that major differences exist in the impact of COVID-19 on health outcomes by gender, minoritized student status, first-generation status, and LGBTQ status (8–11), examining heterogeneity in alcohol use patterns across various college student subgroups amidst COVID-19 remains important. For example, increases in alcohol frequency, heavy drinking, and alcohol-related harms were observed only in women (12), and the link between psychological distress and alcohol use and use severity was observed only in women (13,14). Lastly, during the pandemic sexual/gender minority individuals have been at disproportionate risk for mental health concerns and substance use (15,16).”

2. *It would be interesting to describe how the situation was in 2021 when the study was implemented. There are countries, such as Spain, where the measures and policies were very strict by then (curfews, limitations on being at the street, limitations on group reunions, etc.). These specific situations may have had an impact on the alcohol use tendencies in college students. If students had to share more time in campus because of some specific policy, maybe it led to a higher sense of belonging, and vice versa. For example, during 2021, in some Spanish universities, students only had to be one of every three weeks present in classes, with the 2 other weeks following remote lessons. This situation irremediably must affect the sense of belonging, particularly in those that 2021 was their first college year. The authors include some reflections in the discussion about this issue, but I feel that it would be positive to address it in the introduction also.*

We thank the reviewer for this suggestion and have now added two additional sentences in the “Current Study” section of the introduction to specify the conditions in this campus. We include it here for quick reference: “Additionally, by Spring 2021, when this study was conducted, this institution had transitioned back to pre-COVID teaching modalities. The campus was open, and almost all classes had returned to in-person formats.”

3. *Method and analyses. It is no[t] (sic) clear for me if the research questions included where or with whom the participants drank, because it is not the same to drink alone that to meet some friends or colleagues to have some drinks. I believe that this is also an interesting point to consider in this investigation, because I assume that the way and the people you drink with affect in any way to the sense of belonging.*

We thank the reviewer for this thoughtful question. Even though we had included a question in the survey regarding “with whom” the participants drank alcohol (i.e., answers coded as “with others” or “alone”), the base rate of students who drank alone was extremely low in this sample (Person level *N* = 4). Hence, it did not make sense to use this outcome measure in our analysis even though we considered this earlier given the theoretical interest in assessing potential linkages between sense of belonging and isolation; and whether it leads to more solitary drinking behavior vis-a-vis social drinking. Future research should include analysis of whether belonging and alcohol use linkages vary by such social contexts in college—ie., social drinking or solitary drinking.

4. *Discussion. Also, although the discussion is very well conducted, and the analyses show interesting results, I got confused during the reading of the manuscript because I do not know if the results were as expected or were completely different. I know that the research questions were mostly descriptive, and that the study design responds to them, but for me was unexpected the result “a higher uncertainty in belonging is related to a less likely of consumption of alcohol”. It is true that the authors explain this result in the discussion, but in the introduction, I could not find information related to this possible result, or to the contrary. In fact, it seems that the main result (that URM population had the opposite behavior than the other groups) has nothing to do with the objective of the study (because it is no mentioned that this result can be achieved).*

We have now revised the introduction and discussion sections to further highlight this study’s novel contributions. Given the limited number of studies that have examined college students’ sense of belonging with their institution and alcohol-use (see Neighbors et al., 2021 for an exception), we have been careful to note, wherever possible, whether our findings replicated prior work or showed mixed/new results.

- For example, see second paragraph in our discussion in this version: “First, we found that on days when students felt more uncertain about their belonging in college than usual (i.e., controlling for person-level belonging uncertainty, which is also negatively related to drinking behaviors), they were less likely to drink and consumed fewer drinks. This negative association might be a result of students seeking out fewer social interactions on days that they feel like they belong less, given that drinking is a very social activity on college campuses. These findings are in line with prior patterns observed in the literature from cross-sectional snapshots that higher levels of belonging might indeed be a risk factor for engaging in drinking behaviors (33).”
- We then highlight the heterogeneity observed among minoritized students. We recruited a large sample size and collected key sociodemographic factors such as race/ethnicity, first-generation college status, and LGBTQ status, deliberately to examine variation and heterogeneity among key health disparity groups, based on extensive prior theoretical and empirical work highlighting the need to understand risk and resilience factors related to alcohol use among these subgroups. So, this was very much a core objective of our study. We highlight those results next in the discussion: “Next, and most concerningly, we observe that on days that minoritized students feel more uncertain about their belonging, they are more likely to drink. Indeed, we see that the sign and strength of the associations for students from these health disparity populations is significantly different from the full sample results—pointing to the need to examine heterogeneity across important interpersonal dimensions (42). The sensitivity of the relationship between students’ uncertainty about their belonging with their institutions and the likelihood of drinking is a particularly concerning moderation, likely exacerbated during the pandemic. Although we interpret this effect with caution given sample size differences across various minoritized subgroups, we believe these trends warrant further attention that future research from more diverse, large, samples should focus on.”
- Lastly, we also provide new insights on the dynamic relationships in our key predictor variable—sense of belonging—among health disparity populations and discuss how these findings line up (or not) with prior research. See here “Finally, minoritized, first-generation, and LGBTQ students show slightly higher belonging uncertainty even on a day-to-day basis, largely replicating prior work (19,23). We also observe larger within-person variations in belonging uncertainty for these groups. These results are consistent with prior theory and empirical research, that minoritized students, especially in predominantly white institutions are often exposed to daily slights and adversities that make them question their day-to-day belonging (Walton & Cohen; 2007; 2011). Our study documents that larger variations are also found among other health-disparity populations—such as LGBTQ students—that warrants further research.”

5. *It is clear that alcohol consumption can be problematic in university students, and that university institutions must address this issue; however, I believe that the future lines of investigation are also scarce. There is potential in the study results that I believe is not being addressed. On the one hand, to implement measures of welcome, of belonging that do not involve alcohol consumption (since, according to the study, the greater the feeling of belonging, the greater the alcohol consumption). On the other hand, what can be done with minority populations (who happen to be the ones who consume more alcohol when they feel less belonging)? For all these reasons, I consider that the article should be rewritten, emphasizing in the introduction the different points that I have tried to highlight in the review.*

We agree that the implications of this research are complex and nuanced. We have now tried to incorporate all these conundrums and implications in the revised “Discussion” section.

- The last paragraph of our discussion section that addresses this concern is included here: “Campus closure, stricter norms of gathering during the pandemic, etc. could have contributed to the overall low levels of social drinking that we might be capturing here. That said, students reporting high belonging uncertainty (i.e., lower belonging) may have had fewer opportunities to socialize with others, when drinking is more common and therefore drink less. This interpretation highlighted by Neighbors et al. (2021) in their study aligns somewhat with our study findings. In other words, lower likelihood of drinking and drinking less on days when students feel more uncertain about their belonging, are positive outcomes overall given the college population we are studying. However, if these are proxies for social isolation, especially for students from historically, marginalized sociodemographic subgroups in college, future research and outreach/institutional programming efforts must pay close attention to these patterns.”
- Our revised conclusion section also addresses this concern now: “Our findings point towards the continued need for more data and research from a variety of institutions as students continue to navigate their college experiences amidst the pandemic. Several studies have found that students from minoritized backgrounds often report lower levels of belonging on days when they experience negative, interpersonal interactions. Interventions that provide more adaptive interpretations of struggles during the transition to college, have buffered students from making global assessments about their sense of belonging, which results in a self-reinforcing cycle of positive outcomes for those students (19,21). Such interventions can be further adapted to reduce the likelihood of increased drinking as a strategy for gaining acceptance or feeling more connected.”

# ***Responses to Comments by Reviewer 3***

*Overall, the manuscript provides valuable insights into the linkages between a key psychosocial factor—students’ sense of belonging in college—and their alcohol use patterns amidst COVID. The manuscript's findings hold significant value for mental health care and public health services. It is well-written and organized, with a clear introduction that sets the context for the study. However, implementing the recommendations would further improve the clarity, robustness, and applicability of the findings.*

We thank Reviewer #3 for this encouraging feedback!

Below we discuss how we address the remaining recommendations made by Reviewer #3

1. *General recommendation. Although the language is generally clear, improving sentence structure, selecting better words, and ensuring coherence could enhance readability. Additionally, it is recommended to proofread for grammatical and typographical errors*

We have carefully reviewed the manuscript for typos, as well as appropriate grammar and syntax.

2. *The reference style does not meet the journal's requirements, and it should adhere to the Vancouver style.*

We have now converted the reference style from APA to the Vancouver style to meet the journal’s requirements.

3. *More specific recommendations. Introduction - The introduction is well-written, concise, and clear, effectively highlighting the gap in the literature and explaining the aim of the present study. The heading for the current study at the end of the introduction is provided very effectively.*

We thank the reviewer for this thoughtful engagement with our work!

4. *Method. In this section, it is advisable to introduce the study's methodology before delving into the sampling and population. This could include specifying whether the study is qualitative or quantitative, descriptive or applied, and whether it involves multivariate correlation analysis, among other relevant details*.

We agree! We have now added a sentence that specifies the use of a 21-day daily diary survey and quantitative, longitudinal analysis using mixed models to answer our research questions (see page 7) before delving into the “participants” and “procedures” subsections in the “Method” section overall.

5. *Participant’s part is clear, however:*

- *Table 1, labeled as "Descriptive Statistics" in this section, should be renamed to avoid misleading the reader. This is because Table 2, titled as "Descriptive Statistics of Participants," also appears in the results section, and it's important to distinguish between the two.*

We agree! We have now renamed all the tables. Please see below comment #6 for the revised table titles.

- *The paragraph that begins with "In all, 2,068 participants consented and participated in the Student Engagement…" should be titled as "Procedure" as a subheading in the Method section.*

We agree! We have now added a subheading titled “Procedure” within the “Method” section. This section includes the paragraph that begins with “In all, 2068 participants…” and the one that begins with “If students were eligible, the survey…” as that paragraph also described the procedure for survey completion.

6. *Results. The results section offers a comprehensive presentation of relevant statistical analyses. The tables effectively convey important data necessary for interpreting the results, and the author skillfully avoids redundancy and unnecessary elaboration within the tables. However, the titles of the tables need to be more specific and clear for readers.*

We thank the reviewer for this suggestion. We have now renamed all the table titles to make it more specific and clearer for the readers. We include the revised table titles below here for quick reference:

Table 1. Descriptive Statistics of Analytic Sample

Table 2. Means and Standard Deviations of Key Variables (Disaggregated by Demographic Subgroups)

Table 3. Mixed Models Examining Associations between Belonging Uncertainty and Alcohol Use Behaviors

7. *Discussion. The discussion part effectively explores the study findings and comparing them with previous research. It appropriately addresses the limitations of the study and suggests avenues for future research. It is recommended that the last paragraph of the discussion part be titled "Conclusion" to provide clarity for readers. In the last sentence of paragraph 2, which starts with "These findings are in line with prior patterns observed in the literature from cross-sectional…", the reference is missing.*

We thank the reviewer for this suggestion and have now titled the last paragraph of the Discussion section in the prior version beginning: “ Our findings point towards….” as “Conclusion” in this revised version. We also thank the reviewer for catching this oversight regarding missing references. We have now added the references requested at the conclusion of the second paragraph of the discussion (see page 21).

# ***Responses to Comments by Reviewer 4***

*I am so grateful to had an opportunity to read this valuable manuscript. After a detailed review of the submitted manuscript, below are some notes that may help improve readers' understanding.*

We thank the reviewer for their thoughtful review and feedback on our manuscript. Below we include details regarding how we addressed Reviewer 4’s specific comments:

1. *It is suggested to mention the sampling method.*

A random sample of participants who met all the eligibility criteria (1st through 4th-year full-time, undergraduate, college students aged 18-24 years) were recruited to the study through email invitations. We have now added this sampling method in the “Participants” section.

2. *What exclusion criteria were considered?*

Other than the above eligibility criteria (1st through 4th-year full-time, undergraduate, college student aged 18-24 years), no other exclusion criteria were considered. We now explicitly state this in page 7 in the “Participants” section.

3. *It is suggested to briefly mention ethical considerations and how to obtain consent*.

We thank the reviewer for this suggestion and include details regarding the “informed consent” procedure and all other ethical considerations involved in the current study that the university’s IRB approved (see pages 8-10).

4. *Were psychometric criteria such as face validity considered before choosing the question to measure the sense of belonging? How are you sure that the said question has the ability to measure this concept in the population?*

The measure of sense of belonging (i.e., belonging uncertainty we used in the current study) has been extensively studied in the college student sample that bolsters the face validity of the measure. Please see (Gopalan et al., 2022; Murphy et al., 2020; Walton & Cohen, 2007; Yeager et al., 2016). We have included these citations throughout the manuscript as well as specifically in the “measures” section. That said, we also note the limitations of using a single-item measure of belonging uncertainty in the current study in the “limitations” section. We also highlight the continued need to understand the measurement challenges—such as psychometric criteria and validity—for understanding students’ psychosocial experiences such as belonging with a relevant citation in this revised version (see page 19).

5. *The second paragraph of the discussion needs some refrences.*

We thank the reviewer for catching this oversight. We have now added the references requested at the conclusion of the second paragraph of the discussion (see page 17).

6. *In the title of the article, it is mentioned that the study was conducted during the Covid pandemic, but there is not much mention of the role of Covid in the level of fear, anxiety and isolation of people, and especially in the discussion part, there is not much mention of it and similar studies.*

In the first paragraph of the “Discussion” section, we mention the role of COVID on college students’ mental health outcomes and belonging. For example, the three studies we cited (Giuntella et al., 2021; Gopalan et al., 2022; Parchem et al., 2021) all pertain to the potential role COVID played in affecting college students’ psychological experiences. Additionally, we also briefly discuss the role of campus closures during the pandemic as a likely factor for overall lower levels of drinking among our college student sample in page 18. We hope that the above citations and studies examining the role of COVID in affecting the experiences of the college student population in our study and how it provides an important context within which our study results should be interpreted is now clearer in this version of the manuscript.

# ***Responses to Comments by Reviewer 5***

1. *An operational definition of 'belonging' could be included. The authors may qualify "public university in the Northeast" of so-and-so country under participant inclusion criteria.*

The operational definition of “belonging” used in the current study is included in page 10 of the manuscript within the “Methods” section. However, if the reviewer has suggestions/requests pertaining to what they mean here, we will be happy to add that to the manuscript. We have added the country (United States) now in the participant inclusion criteria.

2. *It was not very clear whether the authors intended to and/or found drinking alcohol to be indicative of belonging, or if drinking was a coping mechanism for belonging uncertainty*.

Given the nature of the study (i.e., daily diary study that assessed both drinking behaviors and belonging uncertainty measures at the same points of time each day), it is quite hard to assess the exact directionality, reason/mechanism driving the association between belonging and drinking behaviors. While it is possible that participants might engage in drinking alcohol to fulfil their need to belong (i.e., more uncertain about their belonging) in college given how drinking in college is a very social activity, it could simultaneously be a coping mechanism that could result in isolation and more lack of social integration in campus. We highlight this both in the “limitation” section (see page 20) and need for future research to disentangle these relationships in the “discussion” section (see page 19).
