# Supplementary material for: College students’ sense of belonging and alcohol use amidst COVID-19: Evidence from a 21-day daily diary study
Source: PLoS One. 2024 Dec 3;19(12):e0310496. doi: 10.1371/journal.pone.0310496 (PMC11614200; doi:10.1371/journal.pone.0310496)
Supplement: Attachment — Submitted filename: ResponseLetter_R2.docx [file pone.0310496.s006.docx]

June 25, 2024

Dear Editors:

Thank you for your invitation to resubmit our manuscript, “College Students’ Sense of Belonging and Alcohol Use amidst COVID: Evidence from a 21-day Daily Diary Study”. We are grateful to you and the reviewers for your thoughtful consideration of our work and your helpful suggestions. We believe that the revision fully addresses all concerns raised and is a stronger manuscript. We are not clear what Reviewer #5’s concerns are referring to in this version. So, please let us know if we can do anything more to address those.

In the sections that follow, we respond to each reviewer’s concerns (shown in italics) in turn. In our responses, we indicate the specific changes we made and, as appropriate, point to relevant pages and tables; we have also used track changes in the revised version of the manuscript.

Again, we are grateful for the opportunity to resubmit a revised version of this manuscript to *PLOS ONE*. We look forward to your response.

Sincerely,

Authors

# ***Responses to Comments by Reviewer 1***

*Overall, this revised version of this manuscript addressed correctly most of the comments provided by reviewers. I want to thank the authors for their work, their thorough responses to comments, and I believe they improved greatly the quality of this manuscript. However, some modifications are still needed as some aspects have not totally been addressed and some small modifications are needed to ease reading flow and overall comprehension.*

**We thank Reviewer # 1 for thoughtfully engaging with our work.**

1. *I did not identify this earlier, but I believe that the right way to state the COVID pandemic is to specify the COVID-19 pandemic as coronaviruses were present before 2020 and induced epidemics before. Therefore, to properly identify this work in its historical context, it is needed to add the -19 as it refers to the year 2019.*

**We have now revised to refer to the pandemic as COVID-19 throughout.**

2. *Abstract: This sentence in the result section is long and unclear: “Results revealed that on days when students felt more uncertain about their belonging (i.e., low belonging) with their colleges, they were less likely to drink, and drink less overall even if they do when back on campus after pandemic-related college closures ended.” Please change the phrasing to clarify it’s meaning. Here is a suggestion: Results revealed that on days when students felt more uncertainty about their sense of belonging to their college (i.e., low belonging), they were less likely to drink, and drink less overall. This effect was also present when students went back on campus after pandemic-related college closures ended indicating a limited effect of measures taken to curb the spread of COVID-19 on the relation between sense of belonging and alcohol consumption.*

**We have made the suggested changes with some minor edits to the “results section” in the abstract to improve clarity. “Results revealed that on days when students felt more uncertainty about their sense of belonging to their college (i.e., low belonging), they were less likely to drink, and drink less overall. This effect was observed after students were back on campus after pandemic-related college closures ended.” We have not included the last sentence as our study did not analyze effects of any pandemic-related measures per se to curb the spread of pandemic or other measures related to alcohol consumption. Therefore, our study cannot speak to such effects.**

3. *Introduction: Overall, this section has been dramatically improved. I appreciate the inclusion of clarifications regarding sense of belonging, as well as inclusion of more detailed presentation of the relevance of including sexual diversity, gender diversity, and racial diversity in your paper. Further comments are only superficial aspects to ease reading flow and clarification for potential lay audiences.*
   a. *Please add some context in the introduction, especially when using references that are specific to the USA. For example, 4-year universities are a US-specific form of post-secondary education structure that are not necessarily present in other countries.*

**We have changed four-year university to public, postsecondary university (see page 8)**

b. *Furthermore, what do you mean by “[…] highest rates of drinking”? Are these students more prone to drinking in general? I suggest rephrasing this to reflect the USA-specific status of the study cited for this sentence and clarify what is meant by highest rates of drinking.*

**We have added clarity to this sentence. “For example, 19/20-year-olds who currently attend postsecondary universities and do not live with their parents exhibit the highest rates of drinking among adults in the US.”**

c. *Please identify typos and add commas when necessary. For example, this sentence needs a comma after pandemic: “Lastly, during the pandemic individuals who identify as LGBTQ+ have been at disproportionate risk for mental health concerns and substance use (15,16).”*

**Thank you for your keen eye. We have added a comma to this sentence now. We have also scrubbed the whole text for additional typos/grammatical errors such as the one above.**

d. *This sentence looks like the long dash is used as a parenthesis: “On the prevention side, a key emerging protective factor against adverse mental health outcomes—such as anxiety and depression, which often co-occur with alcohol use (e.g., 17), was students’ sense of belonging with their university (18).” If so, please close it. Some quote marks are not closed: (e.g., “I feel like I belong at [college]; “How […]).*

**We have revised the em-dash to a comma in this sentence to improve clarity. We have also checked that all quotation marks have now been closed.**

4. *Methods: Here, also, most comments have been properly addressed or responded. However, two aspects still need to be addressed as they have been partially corrected.*
   a. *I still believe that descriptive statistics of the sample (as presented in table 1) is a result per se and therefore belongs in the results section. Methods section should only contain information on the questionnaires/measures taken and superficial information on the participants such as recruitment procedure, criteria of inclusion and exclusion to address the “How do you respond to your research question?”. Furthermore, the second paragraph of the “procedure” section, especially starting at: “While the analytical sample […]” also is a presentation of results and do not reflect the recruitment procedure nor methods. I’ll let the authors decide if they want to change this or not.*

**We have now moved Table 1 as well as any text in the *Procedure* that described the sample/descriptive statistics to the “results” section.**

b. *You addressed partially my comment regarding the LGBTQ question but did not clarify how this status was measured in the first place. Is it a simple binary question “Do you identify as LGBTQ? Yes/No” or was it measured differently? I appreciate the addition of a statement in the discussion section, but this is still not clear in the methods as you still do not clearly state how the measures are taken, nor it is detailed in the table 1. Again, as stated in my previous review, I could understand and agree with such a grouped classification, but your LGBTQ sample is not small with almost 17% of the sample and sexual/gender diversity are different concepts that could present very different realities. Therefore, more justification is needed here if the initial measure was not a binary question. As race/ethnicity is presented in a more stratified fashion, I want to be able to see which aspect of the LGBTQ acronym is so small that you need to group them together. Furthermore, maybe a stratification splitting sexual diversity vs gender diversity could be relevant to reflect and address the heterogeneity in the LGBTQ acronym.*

**In the original study (SELF protocol), two questions were asked, one about gender identity and another about sexual identity. However, given privacy concerns, in our study’s IRB protocol, we only requested data on a calculated variable “LGBTQ – Yes/No” from the larger study protocol. So, we do not have the ability to split this group for further analysis in this study. We have now added this as an additional footnote in the “Measures” section.**

**“Even though the SELF protocol collected information on gender identity and sexual identity in two separate questions, we only have a calculated variable on gender/sexual minority (LGBTQ – yes/no) in our analysis, as approved by this study’s IRB”**

5. *Results: No problem here.*
6. *Discussion/conclusion: In this section, comments have been mostly addressed too but slight modifications are required.*
   a. *I believe the limitations section should be before the conclusion as the conclusion is supposed to include all aspects of the article, whether positive or negative. Furthermore, the conclusion should be updated to reflect identified limitations and englobe everything. No need to expand on limitations but a quick sentence acknowledging them and counterbalance it with the strong aspects of your work would be sufficient.*

**We have made the requested change. We just retained the last paragraph in the prior “limitations” section in the Conclusion section in this version to acknowledge and counterbalance it with the main contributions of our study.**

b. *I appreciate the inclusion of identified limitations. However, I will reiterate the fact that aggregation by LGBTQ status is not justified enough here, and the limitation do not address in detail the differences inherent to the large umbrella term of LGBTQ. A simple statement acknowledging this might be sufficient, but it is still needed here.*

**We have now added this sentence in the limitations section to acknowledge this further. “Third, despite our large sample size (both person- and day-level) overall in the analytic sample, disaggregation by finer-grained race/ethnic, or LGBTQ subgroups is constrained by statistical power. For example, the LGBTQ categorization might mask potential differences by gender- and/or sexual-identity categories that we have not been able to tease out adequately.”**

c. *I appreciate that you addressed my comment about controlling for the day of the week and added a table to reflect this but maybe adding a little sentence in the results/discussion section could help readers identify faster that you addressed this question already. I saw the note under the Table 3, but it is rather discrete. Furthermore, why using Sunday as the reference? Just a small rationale justifying this choice would be enough I believe.*

**We moved the “note” to the main ms to ensure its not discrete. Again, the day of reference choice was completely arbitrary, and results do not differ as they are flexibly included in the models. We now include this sentence in page 16 to clarify these choices. “Results are similar when day of week is controlled for in these models, and available in the appendix (see Table A2). The reference day used (i.e., Sunday vs. other days of the week) in the models also do not affect the results (results available upon request).”**

d. *I realized that appendix table A1 has no title. Please add one.*

**Thank you for pointing this out. We have added the title to Table A1 now.**

# ***Responses to Comments by Reviewer 2***

*I thank the authors for their efforts to improve their article and to answer and discuss all the issues I raised in the first review.*

**We thank Reviewer #2 for thoughtfully engaging with our work.**

# ***Responses to Comments by Reviewer 3***

*Appreciate to the author for incorporating all the revisions based on the reviewer's comments and providing explanations for each change. The revised manuscript demonstrates improved design and writing, with a clear and cohesive introduction and discussion section. Overall, the revised manuscript meets the criteria for acceptance.*

**We thank Reviewer #3 for thoughtfully engaging with our work.**

# ***Responses to Comments by Reviewer 4***

*No comments*

# ***Responses to Comments by Reviewer 5***

*The authors have not adequately addressed the comments raised in the previous round of review. I request the authors to clearly state things like definitions, sources, references, citations, and so forth that other reviewers and I have suggested. Otherwise, it is difficult to okay the submission for publication.*

**We adequately addressed R5’s comments from the prior round. We have included below their comments and our response to their comments for your reference. So, we are not sure what his comments on this version refers to when saying “I request the authors to clearly state things like definitions, sources, references, citations, and so forth that other reviewers and I have suggested”. We are happy to provide additional clarifications if the ACADEMIC EDITOR can provide additional clarity on R5’s concerns here.**

1. *An operational definition of 'belonging' could be included. The authors may qualify "public university in the Northeast" of so-and-so country under participant inclusion criteria.*

**The operational definition of “belonging” used in the current study is included in page 10 of the manuscript within the “Methods” section. However, if the reviewer has suggestions/requests pertaining to what they mean here, we will be happy to add that to the manuscript. We have added the country (United States) now in the participant inclusion criteria.**

2. *It was not very clear whether the authors intended to and/or found drinking alcohol to be indicative of belonging, or if drinking was a coping mechanism for belonging uncertainty*.

**Given the nature of the study (i.e., daily diary study that assessed both drinking behaviors and belonging uncertainty measures at the same points of time each day), it is quite hard to assess the exact directionality, reason/mechanism driving the association between belonging and drinking behaviors. While it is possible that participants might engage in drinking alcohol to fulfil their need to belong (i.e., more uncertain about their belonging) in college given how drinking in college is a very social activity, it could simultaneously be a coping mechanism that could result in isolation and more lack of social integration in campus. We highlight this both in the “limitation” section (see page 20) and need for future research to disentangle these relationships in the “discussion” section (see page 19). We conducted this study based on past literature on belonging which was the main focus of the study.**
